# The Unwelcome Guest: *Strongyloides stercoralis* Hyperinfection in a Patient With Steroid-Dependent Asthma–COPD Overlap Syndrome (ACOS)—A Case Report and Review of Literature

**DOI:** 10.1155/crpu/3204304

**Published:** 2025-03-19

**Authors:** Rafael Miret, Jose M. Acosta-Rullan, Alfredo Toll, Grayson Honeycutt, Manjot Malhi, Christian Almanzar Zorrilla, Raiko Diaz, Mauricio Danckers, Daniel Zapata

**Affiliations:** ^1^Department of Pulmonary Medicine and Critical Care, HCA Florida Aventura Hospital, Aventura, Florida, USA; ^2^Department of Pulmonary Medicine and Critical Care, HCA Florida Kendall Hospital, Kendall, Florida, USA

## Abstract

*Strongyloides stercoralis* is a soil-transmitted roundworm nematode estimated to affect over 600 million people worldwide. Hyperinfection syndrome (HS) has been described in immunosuppressed patients. Our case highlights a rare manifestation of HS due to *Strongyloides stercoralis* causing acute respiratory failure in an asthma–COPD overlap syndrome (ACOS) patient on chronic corticosteroid therapy. A 63-year-old woman with diabetes, chronic obstructive pulmonary disorder due to chronic cigarette smoking, and severe asthma on chronic prednisone therapy presented with recurrent intractable abdominal pain and shortness of breath. The patient underwent esophagogastroduodenoscopy (EGD) showing friable mucosa returning positive for *Strongyloides stercoralis* infection. The patient deteriorated with progressive acute hypoxic respiratory failure and acute metabolic encephalopathy requiring invasive mechanical ventilation. Dual antiparasitic therapy with ivermectin and albendazole was initiated, and the patient was treated for septic shock. The patient was successfully extubated and was discharged from the hospital to a rehabilitation center without steroid therapy. Due to the classic transmission and life cycle of the filiform larvae, the lungs are target organs in HS. The mortality of *Strongyloides* HS ranges from 85% to 100% when untreated. HS due to *Strongyloides stercoralis* carries a high risk for disseminated infection in patients with chronic steroids. High index of suspicion, tissue sample, and prompt institution of target therapy institutions are key for a successful clinical outcome.

## 1. Introduction

Strongyloidiasis is well known to be caused by the female nematode *Strongyloides stercoralis* [1–3]. This parasitic infection has been reported to affect around 600 million people worldwide, especially in areas with suboptimal sanitation [4]. The roundworm can be seen in subtropical places and is endemic to the Southeast part of the United States, as seen in our patient. Humans are affected when the parasite penetrates the skin or when it is ingested. The life cycle consists of *Strongyloides* traveling from the skin to the lungs and finally reaching the gastrointestinal (GI) tract of its host [1, 4]. Hyperinfection syndrome caused by this nematode consists of a magnified life cycle and is directly proportional to parasite burden. This can happen in any host; however, the most common risk factor is immunosuppressive state with corticosteroid use being the frequently described [1, 2, 4–7]. We are describing a *Strongyloides stercoralis* hyperinfection in a steroid-dependent patient with ACOS.

## 2. Case Presentation

**Figure 1:**
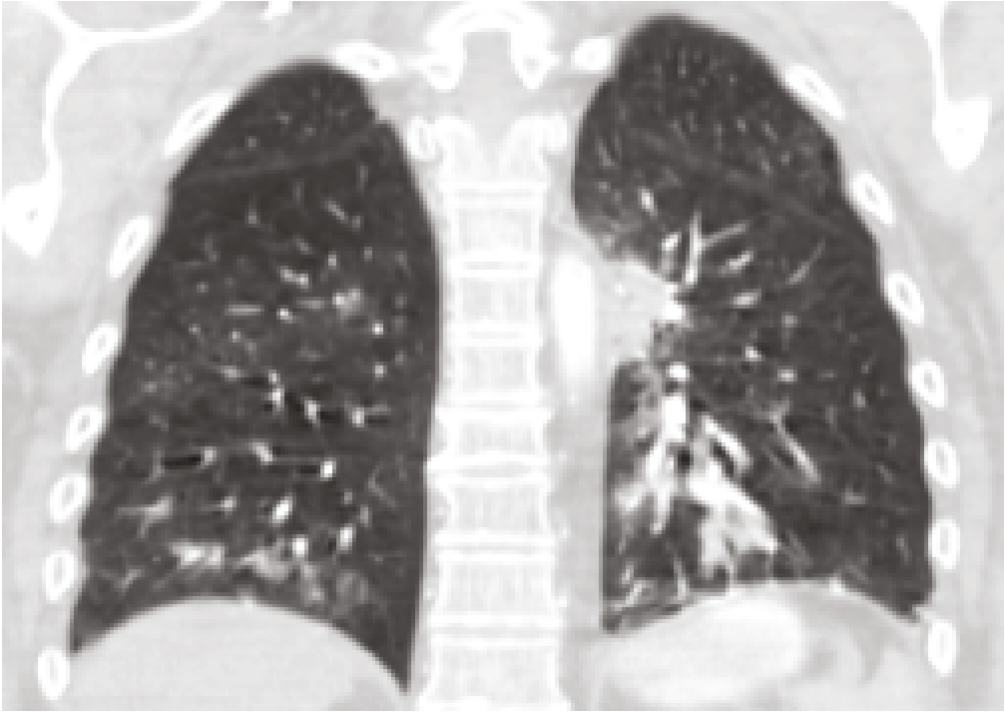
CT scan of the chest showing bilateral ground glass opacities with a left-sided hilar and lower lobe infiltrate.

**Figure 2:**
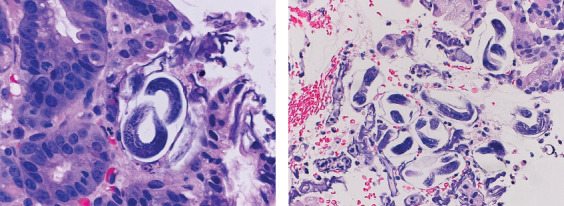
(a, b) Hematoxylin and eosin stain histopathological duodenal biopsy showing *Strongyloides* larvae and eggs.

This is a 63-year-old woman who was born in Cuba with a history of diabetes, chronic obstructive pulmonary disorder (COPD) due to chronic cigarette smoking, and severe asthma on chronic prednisone therapy presented to the emergency department (ED) with recurrent intractable abdominal pain and shortness of breath. The patient had been reporting severe and progressive weakness for the preceding 2 months leading to functional quadriplegia initially attributed to steroid-induced myopathy. Initial blood work revealed hemoglobin of 10.5 g/dL, platelets 480 × 10^3^*  μ*L, sodium of 123 mmol/L, and potassium of 5.1 mmol/L. Chest x-ray (CXR) showed no infiltrates or effusion. Clinical and computed tomography (CT) of the abdomen and pelvis showed mild to gaseous distension of the distal small bowel and transverse colon, mild circumferential thickening of the walls of the distal small bowel, slightly nodular hepatic contour with multiple hepatic hypodense lesions raising concern for gastroenteritis, possible liver abscesses, and no free fluid or air. The patient underwent esophagogastroduodenoscopy (EGD) showing friable mucosa, and gastric mucosa biopsies were obtained. A CT of the chest with intravenous (IV) contrast was obtained and revealed bilateral multifocal ground glass opacities (Figure 1). The patient deteriorated with progressive acute hypoxic respiratory failure and acute metabolic encephalopathy requiring invasive mechanical ventilation. Tissue biopsy demonstrated numerous organisms with morphologic appearance of *Strongyloides stercoralis* in the stomach, incisura angularis, and duodenum (Figure 2a,b). A positive anti-*Strongyloides* IgG (Bordier IVD) was also found.

Dual antiparasitic therapy with ivermectin and albendazole was initiated due to concerns for disseminated disease. Bronchoscopy performed and visualized purulent secretions in the left bronchial tree and bronchoalveolar lavage which was positive for *Pseudomonas aeruginosa* prompting antibiotic coverage. Lumbar puncture was done and noncontributory, and a polymerase chain reaction was not performed. The patient was treated for septic shock with vasoactive medications and IV stress dose hydrocortisone which were weaned over 1 week. The patient was successfully extubated and was discharged from the hospital to a rehabilitation center without steroid therapy.

## 3. Discussion

*Strongyloides stercoralis* have been reported to infect humans, monkeys, dogs, and cats. The filariform larvae penetrate the skin when in contact with contaminated soil. It is a worldwide nematode-transmitted infection, but in the United States, endemic in the Appalachia [8–11]. Based on several studies, *Strongyloides* has a prevalence of 0–6.1% in the United States, with immigrants showing a higher percentage of up to 46% [12].

The life cycle of this parasite consists of two parts: the rhabditiform larvae and the filariform larvae. The rhabditiform larvae are free and live outside the host. During the free cycle, the rhabditiform larvae transform into the infective filariform larvae, which penetrate the skin, then the venous circulation finding their path to the right heart and lungs [1, 3, 13]. When the parasite is residing in the lung, it causes parenchymal distortion leading to alveolar capillary bleeding and, due to vigorous inflammation, eosinophilic pneumonitis [14]. Given this situation, the patients can experience cough, expelling the larvae from the bronchial tree and trachea into the larynx. When reaching the upper airway, they can be swallowed and advance to the stomach and small bowel. In the GI tract, adult female worms can implant themselves in the submucosa and create eggs, via parthenogenesis, and from there, they can be excreted via the stool [1, 2].

The parasite carries the capability of autoinfection. This means that the nematode, by never reaching the soil, can re-enter the host via enteral circulation or perianal skin. This property allows the parasite to remain in the human body for the entire host's life [1–3, 5, 6]. By this feature, when it infects an immunocompromised host, it can advance to fulminant hyperinfection syndrome.

Hyperinfection syndrome occurs mainly in patients with impaired T-cell-mediated immunity, including those with lymphoma, transplant patients, those using corticosteroids, and patients infected with human T-cell lymphotropic virus type 1 [1, 6, 9, 15, 16]. The clinical presentation of this entity is similar to strongyloidiasis; however, due to high parasite load, it can lead to shock, cerebral nervous system involvement, disseminated intravascular coagulation, and respiratory failure like our patient [1, 17]. HS is fatal with profound mortality if not recognized and properly addressed. It is very challenging to diagnose, entailing top-notch clinical judgment. Gram-negative sepsis and meningitis are a known complication due to *Strongyloides* larvae promoting the translocation of *enterica* bacteria into the host circulation.

We aim to highlight this entity in a patient with ACOS due to corticosteroid use. Peripheral eosinophilia is seen in more than 60% of the cases, but only in 20% of patients with HS [9, 18]. It is challenging to think about strongyloidiasis when you encounter a patient with no peripheral eosinophilia due to chronic corticosteroid use. Strongyloidiasis can exacerbate asthma or COPD symptoms, and the treatment consists of steroids which can further exacerbate the disease and lead to HS as described in our patient. This is why we would like to raise awareness, so this syndrome is considered despite not the usual presentation or laboratory findings. The American Society for Transplantation recommends screening patients with epidemiological risk or indecipherable eosinophilia with serology or stool exam. Therefore, screening patients who are at risk due to immunosuppression must be considered [19, 20].

To diagnose strongyloidiasis, the larvae must be visualized under the microscope or PCR of a stool sample is performed. An additional tool can be enzyme-linked immunosorbent assay (ELISA), anti-*Strongyloides* IgG (Bordier IVD) when suspecting this entity. There is no routine recommendation for endoscopy in these patients, but if there are GI manifestations, it can be considered a method to establish the diagnosis, as in our patient.

The cornerstone to treat this condition is ivermectin 200 *μ*g/kg for 1–4 doses depending on the immune status if it is an uncomplicated infection. For severe disease, recommendations are 200 *μ*g/kg daily in conjunction with empiric antibiotic therapy for enteric gram-negative bacteria. There is no specific timeline duration for treatment but the majority of data reports 2-week treatment, until resolution of symptoms and daily stool examination is negative for at least 2 weeks [21]. In patients on immunosuppressive agents, the doses should be reduced.

## 4. Conclusion

This case accentuates a patient with HS without eosinophilia. A high level of suspicion is imperative in these situations, and caution is advised for further steroid administration. Disseminated strongyloidiasis/HS carries an extremely high mortality if not promptly recognized nor treated.

## Data Availability

The authors have nothing to report.

## References

[1] Kassalik M., Mönkemüller K. (2011). Strongyloides stercoralis hyperinfection syndrome and disseminated disease. *Gastroenterología y Hepatología*.

[2] Liu L. X., Weller P. F. (1993). Strongyloidiasis and other intestinal nematode infections. *Infectious Disease Clinics of North America*.

[3] Thompson B. F., Fry L. C., Wells C. D. (2004). The spectrum of GI strongyloidiasis: an endoscopic-pathologic study. *Gastrointestinal Endoscopy*.

[4] Czeresnia J. M., Weiss L. M. (2022). Strongyloides stercoralis. *Lung*.

[5] Roxby A. C., Gottlieb G. S., Limaye A. P. (2009). Strongyloidiasis in transplant patients. *Clinical Infectious Diseases*.

[6] Marcos L. A., Terashima A., Dupont H. L., Gotuzzo E. (2008). Strongyloides hyperinfection syndrome: an emerging global infectious disease. *Transactions of the Royal Society of Tropical Medicine and Hygiene*.

[7] Nutman T. B. (2017). Human infection with Strongyloides stercoralis and other related Strongyloides species. *Parasitology*.

[8] About the Appalachian Region. Appalachian Regional Commission. https://www.arc.gov/about-the-appalachian-region/.

[9] Guerrero-Wooley R., Aranda-Aguirre E., Li W., Wilkin A., Palavecino E. (2017). Case report: Strongyloides stercoralis hyperinfection in a patient with chronic lymphocytic leukemia. *The American Journal of Tropical Medicine and Hygiene*.

[10] Russell E. S., Gray E. B., Marshall R. E. (2014). Prevalence of Strongyloides stercoralis antibodies among a rural Appalachian population—Kentucky, 2013. *The American Journal of Tropical Medicine and Hygiene*.

[11] Starr M. C., Montgomery S. P. (2011). Soil-transmitted helminthiasis in the United States: a systematic review—1940–2010. *The American Journal of Tropical Medicine and Hygiene*.

[12] CDC-Centers for Disease Control, Prevention. CDC-Strongyloides - epidemiology & risk factors. https://www.cdc.gov/parasites/strongyloides/epi.html.

[13] Ganesh S., Cruz R. J. (2011). Strongyloidiasis: a multifaceted disease. *Gastroenterología y Hepatología*.

[14] Akuthota P., Weller P. F. (2012). Eosinophilic pneumonias. *Clinical Microbiology Reviews*.

[15] Siddiqui A. A., Berk S. L. (2001). Diagnosis of Strongyloides stercoralis infection. *Clinical Infectious Diseases*.

[16] Rose C. E., Paciullo C. A., Kelly D. R., Dougherty M. J., Fleckenstein L. L. (2009). Fatal outcome of disseminated strongyloidiasis despite detectable plasma and cerebrospinal levels of orally administered ivermectin. *Journal of Parasitology Research*.

[17] Schaeffer M. W., Buell J. F., Gupta M., Conway G. D., Akhter S. A., Wagoner L. E. (2004). Strongyloides hyperinfection syndrome after heart transplantation: case report and review of the literature. *The Journal of Heart and Lung Transplantation*.

[18] Geri G., Rabbat A., Mayaux J. (2015). Strongyloides stercoralis hyperinfection syndrome: a case series and a review of the literature. *Infection*.

[19] Mejia R., Nutman T. B. (2012). Screening, prevention, and treatment for hyperinfection syndrome and disseminated infections caused by Strongyloides stercoralis. *Current Opinion in Infectious Diseases*.

[20] Schwartz B. S., Mawhorter S. D. (2013). Parasitic infections in solid organ transplantation. *American Journal of Transplantation*.

[21] Henriquez-Camacho C., Gotuzzo E., Echevarria J. (2016). Ivermectin versus albendazole or thiabendazole for Strongyloides stercoralis infection. *Cochrane Database of Systematic Reviews*.

[22] Miret R., Toll A., Aldadah W. (2023). The unwelcome guest: a case of Strongyloides stercoralis hyperinfection in a patient with steroid-dependent asthma- COPD overlap syndrome (ACOS). *Chest*.

